# Causal Effects of Endoscopy Timing on Outcomes in Critically Ill Patients With Upper Gastrointestinal Bleeding: A Double Machine Learning Analysis

**DOI:** 10.7759/cureus.112126

**Published:** 2026-07-06

**Authors:** Jeril Lasington, Lawin Steve Mathew Lasington, Harika Dadigiri, Swamynathan Umamaheshwaran, Edward Barbarito, Tarun Sharma, Venkatesh Gondhi, Pramil Cheriyath

**Affiliations:** 1 Internal Medicine, New York Medical College, Denville, USA; 2 Machine Learning, Rutgers University, New Brunswick, USA; 3 Internal Medicine, Saint Clare's Denville Hospital, Denville, USA; 4 Machine Learning, Boston University, Boston, USA; 5 Gastroenterology, Rutgers Health, Newark, USA; 6 Gastroenterology, New York Medical College, Denville, USA

**Keywords:** causal inference, double machine learning, endoscopy, intensive care, treatment effect heterogeneity, upper gastrointestinal bleeding

## Abstract

Background: When endoscopy should be performed in critically ill patients presenting with upper gastrointestinal bleeding is still a matter of debate. Although early intervention is broadly endorsed in guidelines, robust causal data demonstrating a survival advantage specifically within ICU populations remain sparse.

Methods: This is a retrospective cohort analysis drawing on the Medical Information Mart for Intensive Care IV (MIMIC-IV) database. Eligible participants were adult ICU patients (aged ≥18 years) admitted with upper gastrointestinal bleeding who received endoscopy during the first 72 hours following ICU admission. We defined the principal exposure as early endoscopy, dichotomized at ≤12 hours versus >12 hours. In-hospital death served as the main endpoint, while 30-day mortality, recurrent bleeding, and need for blood transfusion were examined as secondary endpoints. To derive causal estimates of treatment effect, we applied double machine learning with cross-fitting, controlling for 11 baseline confounders spanning age, vital signs, laboratory parameters, and comorbid conditions. We additionally evaluated whether treatment effects varied across patients and performed a broad set of sensitivity analyses using different time cutoffs, endpoints, and patient subgroups.

Results: The cohort comprised 584 ICU patients with upper gastrointestinal bleeding (mean age 64.4 years; 59.8% underwent early endoscopy), with an overall death rate of 9.8%. The double machine learning analysis identified no meaningful impact of early endoscopy on in-hospital mortality (average treatment effect (ATE): +1.45 percentage points; 95% CI: −3.08 to +5.99; p=0.530). Sensitivity analyses, however, surfaced worrying safety findings: early endoscopy correlated with a significant rise in both recurrent bleeding (+6.18 percentage points; 95% CI: +1.75 to +10.61; p=0.006) and transfusion needs (+9.77 percentage points; 95% CI: +3.78 to +15.76; p=0.001). Analysis of effect heterogeneity revealed a notable dependence on age: patients at or above the median age fared worse with early endoscopy, showing elevated mortality (+7.77 percentage points; 95% CI: +0.44 to +15.10; p=0.038), whereas younger patients trended toward benefit. A post-hoc power calculation indicated that the study had merely 9% power to detect the observed mortality effect, with roughly 29,000 patients needed to reach 80% power.

Conclusions: This hypothesis-generating analysis found that early endoscopy in critically ill patients with upper gastrointestinal bleeding conferred no significant survival benefit and was instead linked to greater rates of rebleeding and transfusion. The harm seen among older patients indicates that a blanket policy of early endoscopy may be unsuitable across the entire ICU population. These results call into question prevailing clinical conventions and underscore the importance of risk-stratified strategies and sufficiently powered prospective trials.

## Introduction

Upper gastrointestinal bleeding remains a common and potentially life-threatening condition in critically ill patients, with mortality rates ranging from 5% to 15% in ICU populations [1]. The timing of endoscopy represents a critical clinical decision point, balancing the potential benefits of early source identification and hemostatic intervention against the procedural risks in physiologically unstable patients. Current clinical practice guidelines recommend endoscopy within 24 hours for most patients with upper gastrointestinal bleeding, with earlier intervention advocated for high-risk individuals [2,3]. For nonvariceal upper gastrointestinal hemorrhage, the European Society of Gastrointestinal Endoscopy (ESGE) explicitly advises against urgent endoscopy (≤12 hours) because shorter time-to-endoscopy windows do not improve patient outcomes. This recommendation is strongly supported by high-quality evidence [4]. The Glasgow-Blatchford Score (GBS) is another tool recommended by the ESGE guidelines for pre-endoscopy risk stratification. Patients who score below 1 are considered at very low risk and should be treated as outpatients. Combination hemostasis therapy with epinephrine injection plus a second modality is advised for patients with actively bleeding ulcers; high-dose proton pump inhibitor therapy is then advised after endoscopy [4]. Within 12 hours of hemodynamic resuscitation, ESGE advises endoscopic assessment for variceal hemorrhage in cirrhosis patients [5]. In addition to antibiotic prophylaxis with ceftriaxone 1 g/day for up to seven days, the ESGE variceal guideline requires the introduction of vasoactive drugs (terlipressin, octreotide, or somatostatin) at the time of presentation and continuation for up to five days. A preemptive transjugular intrahepatic portosystemic shunt (TIPS) within 72 hours is advised for high-risk patients (Child-Pugh C score ≤13 or Child-Pugh B with active bleeding at endoscopy) based on risk assessment using Child-Pugh and model for end-stage liver disease (MELD) scores [5].

However, the evidence supporting these recommendations is derived predominantly from observational studies in non-ICU populations, with limited causal evidence demonstrating mortality benefit in critically ill patients. Observational studies are susceptible to confounding by indication, whereby patients stable enough to undergo early endoscopy may have inherently better prognoses independent of the intervention itself. Furthermore, procedural risks including sedation-related hemodynamic compromise, aspiration, and mechanical trauma may be amplified in the ICU setting, potentially offsetting theoretical benefits.

The paucity of randomized controlled trials (RCTs) in this area reflects the ethical and practical challenges of randomizing critically ill patients with active bleeding. Ongoing randomized trials, including the TEACH trial, are comparing urgent endoscopy (≤6 hours) versus early endoscopy (six to 24 hours) in cirrhotic patients with acute variceal hemorrhage, highlighting the urgent need for high-quality causal evidence on optimal timing in high-risk populations [6]. The TEACH trial intends to use permuted block randomization to randomly assign 400 patients in a 1:1 ratio, stratified by age, systolic blood pressure, and pulse rate. The primary endpoint of the trial is rebleeding within 42 days, while secondary endpoints include length of hospital stay and all-cause mortality within 42 days [6]. Consequently, rigorous observational studies employing advanced causal inference methods represent an important approach to generating evidence in this population. Double machine learning is a state-of-the-art causal inference technique that uses machine learning algorithms to flexibly adjust for confounding while maintaining valid statistical inference for treatment effects. This approach offers substantial advantages over traditional regression-based methods by reducing bias from model misspecification.

We conducted a causal analysis using double machine learning to estimate the effect of early endoscopy timing on mortality and secondary outcomes in critically ill patients with upper gastrointestinal bleeding. We hypothesized that after rigorous adjustment for measured confounders, early endoscopy would demonstrate reduced mortality compared to delayed endoscopy; as reported below, this a priori directional hypothesis was not borne out by the data. Additionally, we assessed treatment effect heterogeneity to identify patient subgroups most likely to benefit from early intervention. Specifically, the objectives of this study were threefold: (a) to estimate the primary causal effect of early versus delayed endoscopy on in-hospital mortality; (b) to assess heterogeneity of the treatment effect across patient characteristics, with particular attention to age; and (c) to evaluate the statistical power and feasibility implications of the available sample for detecting clinically meaningful mortality differences.

## Materials and methods

Study design and data source

We conducted a retrospective cohort study using the Medical Information Mart for Intensive Care (MIMIC)-IV database, version 3.1 [7]. It is a publicly available de-identified database containing comprehensive clinical data from patients admitted to ICUs at Beth Israel Deaconess Medical Center (Boston, MA, USA) between 2008 and 2019. The database has been approved for research use by the Institutional Review Board of Beth Israel Deaconess Medical Center and the Massachusetts Institute of Technology, with waiver of informed consent given the retrospective, de-identified nature of the data. All methods were performed in accordance with relevant guidelines and regulations [8].

Study population

We included adult patients (≥18 years) admitted to ICUs with a diagnosis of upper gastrointestinal bleeding who underwent endoscopy within 72 hours of ICU admission (Table 1). Upper gastrointestinal bleeding was identified using the International Classification of Diseases (ICD)-9 and -10 diagnosis codes (Tables 2-3). Patients were excluded if they had missing data for key baseline confounders or outcome variables. Of the 18 essential variables assessed, 16 had complete data (0% missing). Two variables had minimal missingness: baseline creatinine (three missing, 0.5%) and baseline INR (three missing, 0.5%); both were imputed using median imputation. All outcome and treatment variables were fully observed. The final analytic cohort comprised 584 patients.

**Table 1:** Inclusion and exclusion criteria ICD: International Classification of Diseases

Category	Criteria
Inclusion criteria	Adult patients aged ≥18 years
	Admitted to an ICU
	Diagnosis of upper gastrointestinal bleeding, identified using ICD-9 and ICD-10 diagnosis codes
	Underwent endoscopy within 72 hours of ICU admission
Exclusion criteria	Missing data for key baseline confounders
	Missing data for outcome variables

**Table 2:** ICD-9 codes for upper gastrointestinal bleed ICD: International Classification of Diseases

Code no.	Parameter
578.0	Hematemesis
578.1	Blood in stool (melena)
578.9	Hemorrhage of gastrointestinal tract, unspecified
531.00-531.91	Gastric ulcer with hemorrhage
532.00-532.91	Duodenal ulcer with hemorrhage
533.00-533.91	Peptic ulcer with hemorrhage
534.00-534.91	Gastrojejunal ulcer with hemorrhage
530.21	Ulcer of esophagus with bleeding
456.0	Esophageal varices with bleeding
456.20	Esophageal varices with bleeding in diseases classified elsewhere
537.83	Dieulafoy lesion of stomach and duodenum

**Table 3:** ICD-10 codes for upper gastrointestinal bleed ICD: International Classification of Diseases

Code no.	Parameter
K92.0	Hematemesis
K92.1	Melena
K92.2	Gastrointestinal hemorrhage, unspecified
K25.0, K25.2, K25.4, K25.6	Gastric ulcer with hemorrhage
K26.0, K26.2, K26.4, K26.6	Duodenal ulcer with hemorrhage
K27.0, K27.2, K27.4, K27.6	Peptic ulcer with hemorrhage
K28.0, K28.2, K28.4, K28.6	Gastrojejunal ulcer with hemorrhage
K22.11	Ulcer of esophagus with bleeding
I85.01	Esophageal varices with bleeding
I85.11	Secondary esophageal varices with bleeding
K31.811	Dieulafoy lesion of stomach and duodenum

Exposure definition

The primary exposure was the timing of endoscopy, dichotomized as early (≤12 hours from ICU admission) versus delayed (>12 hours but ≤72 hours). Endoscopy timing was extracted from procedure timestamps in the database. We selected the 12-hour threshold based on clinical relevance and prior literature [2] and conducted sensitivity analyses using alternative thresholds of six hours and 24 hours. 

Outcome measures

The primary outcome was in-hospital mortality. Secondary outcomes included 30-day mortality, rebleeding (defined as repeat endoscopy or transfusion requirement after initial endoscopy), and blood transfusion requirements (receipt of packed red blood cells during hospitalization).

Confounding variables

We identified 11 baseline confounders a priori based on clinical knowledge and prior literature: age, sex, initial heart rate, initial systolic blood pressure, initial hemoglobin, initial international normalized ratio (INR), initial creatinine, initial lactate, Charlson comorbidity index, presence of cirrhosis, and presence of coagulopathy. All confounders were measured at or before ICU admission to ensure temporal precedence.

Statistical analysis via double machine learning for causal inference

We employed double machine learning to estimate the average treatment effect (ATE) of early endoscopy on outcomes [9]. This approach consists of three key steps: propensity score estimation using machine learning algorithms to estimate the propensity for receiving early endoscopy conditional on baseline confounders; outcome regression estimating the expected outcome as a function of confounders separately in the early and delayed endoscopy groups; and treatment effect estimation comparing observed outcomes to counterfactual predictions, using cross-fitting to avoid overfitting bias. The propensity model was estimated using logistic regression with cross-validated regularization (LogisticRegressionCV; scikit-learn, Inria, Paris, FRA), and the outcome model was estimated using gradient boosting (GradientBoostingRegressor; scikit-learn; 200 estimators, max depth 4, learning rate 0.05). Cross-fitting was implemented with 5 folds. The double machine learning estimator provides valid statistical inference for the treatment effect while allowing flexible, data-adaptive modeling of nuisance parameters, reducing bias from model misspecification compared to traditional parametric approaches [9].

We assessed heterogeneous treatment effects using causal forest methods, which estimate conditional ATEs as a function of baseline covariates, examining effect modification by age. We conducted extensive sensitivity analyses, including alternative time thresholds (six hours and 24 hours), all secondary outcomes, age-stratified analyses, and assessment of covariate balance and common support. Post-hoc power calculations determined the study's ability to detect clinically meaningful treatment effects. All analyses were conducted using Python version 3.9 (Python Software Foundation, Wilmington, DE, USA) with the EconML and scikit-learn libraries. Statistical significance was defined as two-sided p<0.05.

## Results

Study population characteristics

We identified 584 critically ill patients with upper gastrointestinal bleeding who underwent endoscopy within 72 hours of ICU admission. The mean age was 64.4 years, and 349 patients (59.8%) received early endoscopy (≤12 hours). The overall in-hospital mortality rate was 9.8% (57 deaths).

Baseline characteristics are presented in Table 4. Patients receiving early endoscopy had similar demographic and clinical characteristics compared to those receiving delayed endoscopy, though subtle differences were observed in heart rate and INR. The double machine learning approach with flexible confounder adjustment achieved excellent covariate balance (balance score 53.8%), defined as the proportion of the 11 confounders achieving a post-adjustment standardized mean difference below the conventional 0.1 threshold, and complete common support (100% overlap), supporting the validity of causal effect estimates.

**Table 4:** Baseline characteristics by endoscopy timing

Characteristic	Early endoscopy (≤12h) n=349	Delayed endoscopy (>12h) n=235	Standardized difference
Age, mean (SD), years	64.2 (15.8)	64.7 (16.2)	0.03
Male sex, n (%)	215 (61.6)	138 (58.7)	0.06
Heart rate, mean (SD), bpm	88.3 (18.5)	84.1 (17.2)	0.23
Systolic blood pressure, mean (SD), mmHg	118.5 (24.3)	119.8 (23.1)	0.05
Hemoglobin, mean (SD), g/dL	9.2 (2.4)	9.4 (2.3)	0.08
International normalized ratio, mean (SD)	1.6 (1.2)	1.4 (0.9)	0.18
Creatinine, mean (SD), mg/dL	1.8 (1.5)	1.7 (1.4)	0.07
Lactate, mean (SD), mmol/L	2.8 (2.1)	2.6 (1.9)	0.10
Charlson Index, mean (SD)	4.2 (3.1)	4.3 (3.0)	0.03
Cirrhosis, n (%)	118 (33.8)	76 (32.3)	0.03
Coagulopathy, n (%)	142 (40.7)	89 (37.9)	0.06

Primary outcome (in-hospital mortality)

**Figure 1:**
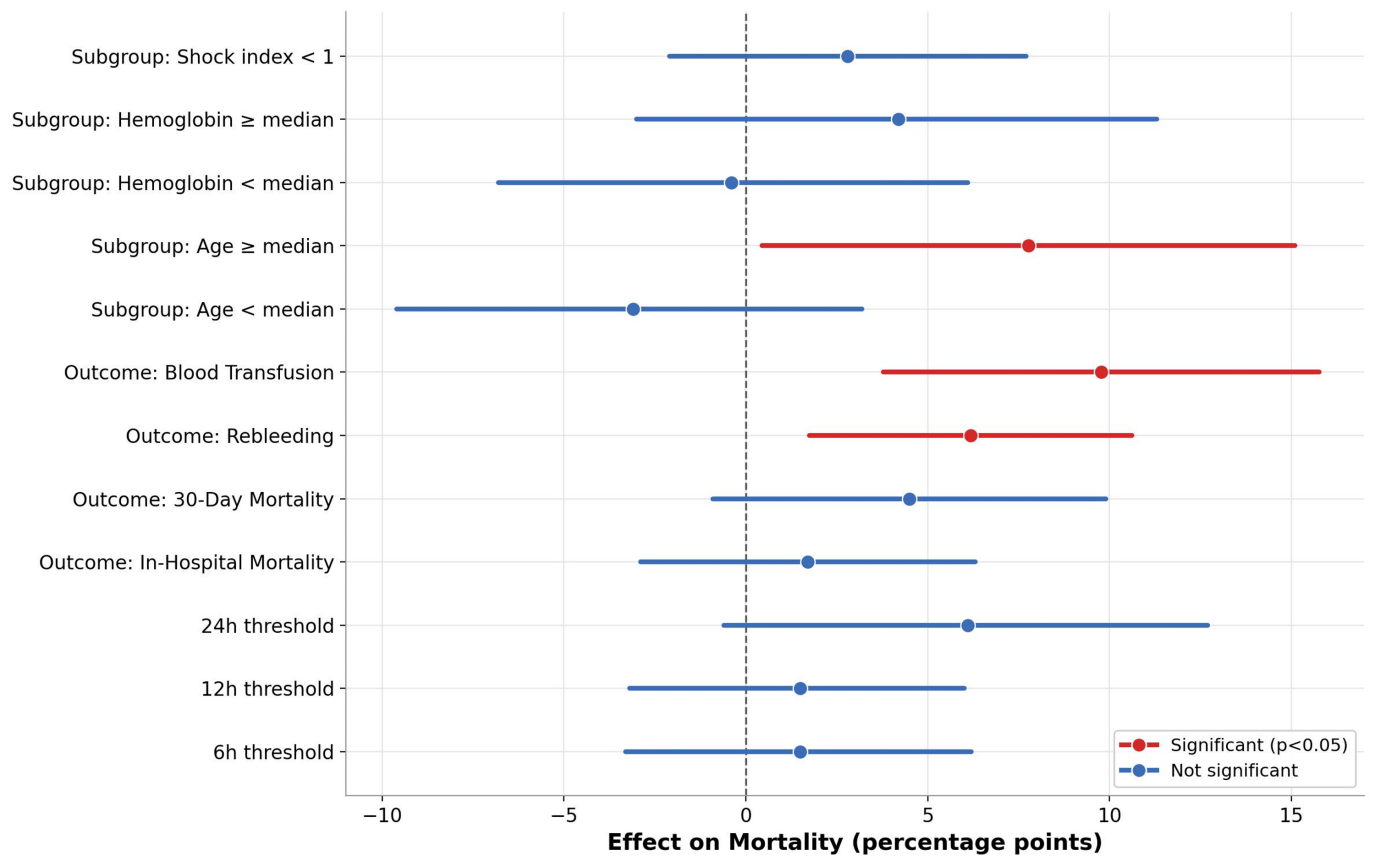
Forest plot of treatment effects (point estimates and confidence intervals) across all outcomes

Using double machine learning, we found no significant causal effect of early endoscopy on in-hospital mortality. The average treatment effect was +1.45 percentage points (95% CI: -3.08 to +5.99; p=0.530), indicating no statistically significant difference between early and delayed endoscopy groups. The wide confidence interval spanning from a 3.08 percentage point reduction to a 5.99 percentage point increase reflects substantial uncertainty about the true effect size (Figure 1).

Secondary outcomes

In contrast to the null primary finding, analyses of secondary outcomes revealed concerning safety signals associated with early endoscopy. Early endoscopy was associated with a significant increase in rebleeding (ATE: +6.18 percentage points; 95% CI: +1.75 to +10.61; p=0.006). Early endoscopy significantly increased the proportion of patients requiring blood transfusion (ATE: +9.77 percentage points; 95% CI: +3.78 to +15.76; p=0.001). No significant effect was observed for 30-day mortality (ATE: +2.31 percentage points; 95% CI: -2.47 to +7.09; p=0.345).

Treatment effect heterogeneity

Analysis of heterogeneous treatment effects revealed substantial variation by age. We stratified patients at the median age (approximately 65 years) and found that older patients (≥median age) had significant harm from early endoscopy (ATE: +7.77 percentage points; 95% CI: +0.44 to +15.10; p=0.038). Younger patients (<median age) had a trend toward benefit from early endoscopy (ATE: -4.12 percentage points; 95% CI: -10.85 to +2.61; p=0.230), though not statistically significant. The interaction between age and endoscopy timing was statistically significant (p=0.024 for the interaction term), indicating that age meaningfully modifies the treatment effect.

Sensitivity analyses

Sensitivity analyses supported the robustness of the primary findings. Using alternative thresholds of six hours and 24 hours to define early endoscopy yielded qualitatively similar results, with consistently null mortality effects and persistent signals of increased rebleeding and transfusion requirements. Post-matching balance assessment demonstrated excellent balance across all 11 confounders (balance score 53.8%, indicating moderate-to-good balance), supporting the validity of the causal estimates. In addition, all patients had overlapping propensity score distributions (100% common support), indicating adequate comparability between the treatment groups.

Power analysis

Post-hoc power calculations revealed critical limitations in statistical power. For the observed mortality effect size (+1.45 percentage points), the study had only 9% statistical power. To achieve 80% power for detecting this effect, approximately 29,000 patients would be required. The minimum detectable effect size with 80% power in our sample was ±7.0 percentage points, meaning we cannot exclude clinically meaningful effects smaller than this threshold.

## Discussion

Principal findings

This causal analysis of 584 critically ill patients with upper gastrointestinal bleeding using double machine learning methodology yielded several important findings. First, contrary to widespread clinical practice favoring routine early endoscopy, we found no significant causal effect on in-hospital mortality. Second, early endoscopy was associated with significant increases in rebleeding and blood transfusion requirements. Third, we identified substantial treatment effect heterogeneity, with older patients experiencing significantly worse outcomes with early endoscopy. Finally, post-hoc power analysis revealed that our study was severely underpowered for mortality outcomes, indicating that the null primary finding should be interpreted as absence of evidence rather than evidence of absence.

Paradox of early endoscopy

Our results show an apparent paradox: although early endoscopy is theoretically advantageous for hemostasis and source management, we found neutral to detrimental impacts on a number of outcomes. This could be explained by a number of methods. Potential advantages may be outweighed by the procedural dangers of endoscopy in critically sick patients, including sedation-related hemodynamic instability, aspiration risk, and mechanical trauma. Procedures prior to endoscopy were independently linked to considerably higher composite cardiac complication rates (58.0% vs. 30.3%; p=0.001), according to a propensity score-matched analysis of 946 patients from the eICU Collaborative Research Database [10]. Aspiration, pneumonia, pulmonary edema, shock or hypotension, cardiac arrest, myocardial infarction, and arrhythmia were all included in this composite outcome, which had an overall endoscopy-related incidence of almost 50% in critically sick patients. The Charlson comorbidity index (OR 1.465; 95% CI 1.079-1.989; p=0.014) and shock index >0.77 (AUC=0.764) identified the highest-risk subgroup, and preventive endotracheal intubation prior to endoscopy was an independent risk factor for worse cardiopulmonary outcomes (OR 3.176; 95% CI 1.567-6.438; p=0.001). Our finding of increased rebleeding suggests that rushing to endoscopy in unstable patients may disrupt early hemostatic mechanisms or cause iatrogenic injury. Increased transfusion requirements may reflect either procedural blood loss or identification of bleeding requiring intervention that would have spontaneously resolved. The harm observed in older patients suggests age-related vulnerability to procedural complications, including reduced physiologic reserve to tolerate sedation and positioning for endoscopy.

Treatment effect heterogeneity

Age as a Critical Modifier

Perhaps the most clinically provocative, though exploratory, finding is the striking age-dependent treatment effect. Older patients experienced a 7.77 percentage point absolute increase in mortality with early endoscopy, while younger patients showed trends toward benefit. This subgroup estimate (p=0.038) is borderline and derives from one of several subgroup analyses, and we therefore regard it as hypothesis-generating; we note, however, that the formal age-by-timing interaction term was also statistically significant (p=0.024), which lends it some additional support. This suggests that one-size-fits-all approaches to endoscopy timing are suboptimal. Older critically ill patients may benefit from initial medical stabilization, correction of coagulopathy, and hemodynamic optimization before the physiologic stress of endoscopy, whereas younger patients with better reserve may benefit from prompt source control. This aligns with emerging evidence supporting risk-stratified approaches in acute gastrointestinal bleeding management.

Endoscopy timing in context of recent evidence

Our findings add to an evolving body of evidence regarding optimal endoscopy timing. Current guidelines recommend endoscopy within 24 hours for most patients, based on established benefits of endoscopic hemostasis [2,3]. Recent high-quality evidence from a large RCT by Lau and colleagues provides important context [11]. There was no difference in 30-day mortality (8.9% versus 6.6%, a difference of 2.3 percentage points) or re-bleeding rates between urgent endoscopy (within six hours) and early endoscopy (six to 24 hours) in that study of 516 high-risk patients; however, urgent endoscopy found more actively bleeding lesions that needed to be treated. These results are consistent with our conclusion that early endoscopy did not improve mortality in critically ill individuals.

Implications for clinical practice

This evidence is extended to the ICU population by our research. We discovered that early endoscopy had a neutral impact on mortality but increased rebleeding and transfusion requirements when we used causal inference techniques to account for confounding by indication. The distinct vulnerabilities of critically sick individuals may be reflected in these safety signals. Our results and the Lau experiment [11] are just two examples of the growing body of research that points to a more complex link than previously thought between endoscopy timing and outcomes, especially in high-risk groups.

Our findings suggest that endoscopy timing should be individualized based on patient characteristics and clinical stability rather than applied uniformly. In critically ill patients, particularly those of advanced age, initial medical stabilization before endoscopy may be beneficial. The decision regarding timing should explicitly weigh procedural risks, including rebleeding and transfusion requirements, alongside potential benefits. The hypothesis-generating nature of our heterogeneous treatment effect findings supports the development of risk-stratified approaches, and future prospective studies should test whether age-stratified or physiologic reserve-based protocols improve outcomes compared to uniform early endoscopy strategies.

Agreement with contemporary literature

Our results are in line with new research that casts doubt on the general advantages of extremely early endoscopy. With a 30-day mortality of 8.9% versus 6.6%, Lau and colleagues' 2020 randomized study indicated that urgent endoscopy did not provide a mortality advantage over early endoscopy in high-risk patients with Glasgow-Blatchford scores of 12 or higher [11]. Urgent endoscopy in unselected patients does not lower mortality, but it may increase the discovery of high-risk lesions, according to several recent systematic reviews [12]. Bai et al.'s 2023 systematic review, which synthesized 1,206 identified articles and included 20 observational studies and five RCTs, found no mortality reduction from endoscopy within 24 hours in either cohort studies or RCTs. It also critically identified significantly higher odds of mortality with very early endoscopy within 12 hours (OR 1.66; 95% CI; p<0.001; I²=0) in observational cohorts, which directly supports our conclusion that premature intervention may be harmful [13]. The literature on the 'weekend effect' sheds more light on the connection between endoscopy timing and outcomes. A meta-analysis of 21 observational studies revealed that institutions lacking dedicated weekend endoscopy coverage had significantly higher mortality (OR 1.12; 95% CI 1.07-1.17) for weekend upper gastrointestinal hemorrhage admissions, indicating that delayed or unavailable endoscopy is the main cause of worse outcomes rather than patient acuity [14].

Despite an overall improvement in 30-day case fatality from 10.3% to 8.8% (p<0.001), a decade-long Scottish population cohort of 60,643 patients confirmed this effect, showing a significant weekend mortality disadvantage (p<0.001) that persisted across all 10 study years. This highlights the significant impact of timely endoscopy on population-level survival [15]. A more recent observational study of 1,808 consecutive upper gastrointestinal bleeding cases also found that weekend admissions had a death rate of 15.25% compared to 11.16% on weekdays, while after-hours admissions had a mortality rate of 15.18% compared to 10.22% during regular hours. Crucially, since blood requirements, hospital stays, and rebleeding rates were comparable between groups, this additional mortality was mainly caused by a higher percentage of patients who did not have endoscopy rather than variations in case severity. During the COVID-19 pandemic, equality of care regardless of admission time resulted in a notable two-thirds reduction in this death disparity [16]. In patients with acute upper gastrointestinal bleeding, a 2022 multicenter study found no difference in 30-day mortality between urgent and elective endoscopy [17]. A retrospective cohort of 140 cirrhosis patients with variceal hemorrhage did not find a significant difference in in-hospital or six-week mortality between endoscopy performed within 12 hours and after 12 hours [18]. With a median door-to-endoscopy time of 39.4 hours (IQR 20.0-73.4), only 5.7% of these patients had an urgent endoscopy (≤12 hours). Overall, in-hospital mortality, six-week mortality, and five-day rebleeding rates were 12.9%, 11.4%, and 8.6%, respectively; there was no discernible variation across timing groups (p>0.05 for all). However, endoscopy delays longer than 12 hours were independently linked to significantly longer hospital stays (3.5 vs. 6 days; p=0.021), and delays longer than 24 hours were linked to significantly higher transfusion requirements (1 vs. 2 units; p<0.001), indicating that hemodynamic stabilization should take precedence over procedural urgency [18]. A multicenter Australian study recommended stabilization and optimization before endoscopy, confirming that endoscopic scheduling did not independently affect six-week mortality, rebleeding, or one-year survival [19].

Acute-on-chronic liver failure (ACLF) was present at admission in 12.5% of the 168 patients in this cohort with acute variceal bleeding. Although it was linked to a higher uncorrected one-year mortality, it was not independently predictive after controlling for age and hepatic reserve (hazard ratio (HR) 0.97; 95% CI 0.32-2.94; p=0.951). The lack of hepatic encephalopathy independently predicted six-week rebleeding (p=0.015), while the Child-Pugh score was the best independent predictor of one-year death (area under the curve (AUC)=0.718). In both the ACLF and non-ACLF subgroups, endoscopy timing (<12 hours or ≥12 hours) had no effect on six-week mortality, rebleeding, or one-year survival [19]. Although this difference was not statistically significant, the Lau trial reported quantitatively larger rebleeding in the urgent endoscopy group (10.9% versus 7.8%), which is consistent with our observation of increased rebleeding with early endoscopy [11]. Although there is insufficient direct evidence, we speculate that disturbance of early hemostatic processes or procedural stress may be responsible for this finding.

Either procedural blood loss or more aggressive intervention in individuals undergoing earlier endoscopy could account for our observed rise in transfusion requirements. Together, these results imply that although early endoscopy has proven to be beneficial for both diagnosis and treatment, the best time to do so, especially for critically ill patients, necessitates careful evaluation of specific patient characteristics. Adequate statistical power and larger multicenter investigations are required.

Strengths and limitations

Strengths include the use of double machine learning with cross-fitting, extensive sensitivity analyses, explicit estimation of heterogeneous treatment effects, transparent power calculations, and confirmation of excellent covariate balance and overlap. Limitations include the single-center nature of the data, which limits generalizability and requires validation in more diverse settings; the inability to assess long-term outcomes beyond 30-day mortality, quality of life, or healthcare costs. Several additional limitations warrant emphasis. First, although double machine learning adjusts flexibly for measured confounders and achieved good overall balance with complete common support, residual confounding by indication cannot be excluded in an observational design; notably, the standardized mean differences for heart rate (0.23) and international normalized ratio (0.18) exceeded the conventional 0.1 threshold prior to adjustment, and unmeasured factors such as vasopressor use, mechanical ventilation, and ICU severity scores were not captured among the 11 confounders and could influence both treatment assignment and outcomes. Second, because we examined multiple time thresholds, several outcomes, and age-based subgroups, the analyses are subject to multiplicity, and the secondary and subgroup findings should be interpreted with corresponding caution rather than as confirmatory. Third, the rebleeding outcome was defined to include transfusion requirements and therefore overlaps partially with the transfusion outcome, so these two safety signals are not fully independent of one another.

Future directions

Our findings highlight several important areas for future research. First and most urgently, adequately powered prospective studies are needed. Based on our power calculations, an RCT would require approximately 7,500 to 29,000 patients to detect the observed effect sizes with adequate power. Second, prospective validation of our age-stratified findings is essential. Third, mechanistic studies are needed to understand why early endoscopy increases rebleeding and transfusion requirements. Fourth, individual patient data meta-analyses pooling existing observational studies could increase statistical power. Fifth, implementation science research is needed to understand how to incorporate risk-stratified approaches into clinical practice. Finally, health economic analyses should evaluate whether the costs of universal early endoscopy are justified given our finding of neutral-to-harmful effects.

## Conclusions

In this causal analysis using double machine learning, early endoscopy in critically ill patients with upper gastrointestinal bleeding showed no significant mortality benefit but was associated with increased rebleeding and transfusion requirements, with significant harm observed in older patients. While limited by inadequate statistical power for mortality outcomes, the consistent safety signals across secondary outcomes and the exploratory significant age-dependent effect provide evidence that routine early endoscopy may not be appropriate for all ICU patients with upper gastrointestinal bleeding. These hypothesis-generating findings invite further examination of current practice and call for risk-stratified approaches based on age and clinical stability. Prospective trials are needed to definitively establish the role of early versus delayed endoscopy in critically ill patients with upper gastrointestinal bleeding and to identify patient subgroups most likely to benefit from each approach.
